# Readiness of Small Energy Markets and Electric Power Grids to Global Health Crises: Lessons From the COVID-19 Pandemic

**DOI:** 10.1109/ACCESS.2020.3008929

**Published:** 2020-07-13

**Authors:** David Carmon, Aviad Navon, Ram Machlev, Juri Belikov, Yoash Levron

**Affiliations:** 1 Israel Electric Corporation Adi 1794000 Israel; 2 Andrew and Erna Viterbi Faculty of Electrical EngineeringTechnion—Israel Institute of Technology 471650 Haifa 3200003 Israel; 3 Department of Software ScienceTallinn University of Technology 54561 12618 Tallinn Estonia

**Keywords:** COVID-19, coronavirus, SARS-CoV-2, health crisis, power system stability, renewable energy, power systems flexibility, energy policy

## Abstract

In this paper we explore how the COVID-19 pandemic, also known as Coronavirus pandemic, affected the operation of small electric grids, and what can this event teach us on the readiness of such grids in the face of future global health crises. We focus on three major effects: changing patterns of generation and consumption, frequency stability, and the joint impact of low consumption and high share of renewable energy sources. Specifically, we analyze changes in consumption in the Israeli, Estonian, and Finnish grids, and attempt to identify patterns of consumption changes that may be explained by the pandemic. We also analyze changes in voltage and frequency, and show that the low consumption caused significant deviations from the nominal values of both parameters. One main conclusion is that the reduced energy consumption during the pandemic is critical, and has a major effect on the operation of small electric grids. Another conclusion is that since the pandemic pushed the relative share of renewable energy to record highs, this event may help us to better understand the influence of a high share of renewables on small grids, thus offering a glance into a renewable-rich future.

## I. Introduction

In December 2019 an outbreak of pneumonia caused by a novel coronavirus occurred in Wuhan, Hubei province, and has spread rapidly throughout China [1]–[3]. After virus identification and isolation, the pathogen for this pneumonia was originally named the 2019 novel coronavirus (2019-nCoV), but has subsequently been officially named the “severe acute respiratory syndrome coronavirus 2” (SARS-CoV-2) by the World Health Organization. On January 30, 2020 the organization declared the 2019 coronavirus disease (COVID-19) to be a public health emergency of international concern [4].

One consequence of the COVID-19 pandemic was a significant change in generation and consumption of electricity worldwide, mostly due to the preventative measures taken by governments [5]. Many countries reported reduced electricity consumption in the commercial and industrial sectors [6]. For instance, the UK normally experiences a 10-20% reduction in electricity demand over the weekend as compared to during the week; however, since the outbreak of the pandemic, the same reduction in consumption was measured during weekdays as well [7]. Another example is the 3% reduction in U.S. demand for electricity on March 27, 2020, as compared to the same day in 2019 [8], [9]. These changes, which are usually rare, affected energy generation and trading, and led to substantial monetary loses in both the public and private sectors [10], [11]. The long-term effects of the pandemic on the energy industry are currently poorly understood, and will be probably investigated for many years to come [12], [13].

In this paper we attempt to better understand how the COVID-19 pandemic affected the operation of small electric grids, and what can this event teach us on the readiness of such grids in the face of future global health crises. We focus on three major effects: changing patterns of generation and consumption, frequency stability, and the joint impact of low consumption and high share of renewable sources. Specifically, we analyze changes in consumption in the Israeli, Estonian, and Finnish grids, both in the time domain and spatial domain, and attempt to identify patterns of consumption changes that may be explained by the pandemic. We also analyze changes in voltage and frequency, and show that the low consumption caused significant deviations from the nominal values of both parameters.

Based on the available data we aim to draw conclusions regarding the readiness of small electric grids to global health crises. One main conclusion is that the reduced energy consumption during the pandemic is critical and has a major effect on the operation of the grid. Therefore, system operators need additional tools to manage events of abnormally low electricity consumption, such as storage devices, enhanced control of variable renewable energy sources, and frequency regulation capabilities. Moreover, we conclude that flexibility is critical, and in many cases long-term reliability should be preferred over immediate economical considerations. We also claim that a high share of renewable energy sources may be a significant factor in the readiness level of small or isolated grids, since such sources may lead to reduced spinning reserves, increased ramp-rates, reduced rotational inertia, and non-optimal economic dispatch. These effects caused by the COVID-19 pandemic may help clarify the influence of a high share of renewables on electrical grids in general, and offer a glance into a renewable-rich future.

## II. The Effects of the COVID-19 Pandemic on Generation and Consumption in Small Grids

The COVID-19 pandemic had a significant effect on generation and consumption of electricity worldwide. In this section we attempt to summarize these effects as they are reflected in three relatively small grids: the Estonian grid, the Finnish grid, and the Israeli grid. Specifically, we analyze changes in consumption both in the time domain and in the spatial domain, and attempt to identify patterns of consumption changes that may be explained by the pandemic. We also show measurements that explain how the reduced consumption affects the voltage profile across the grid.

**FIGURE 1.**
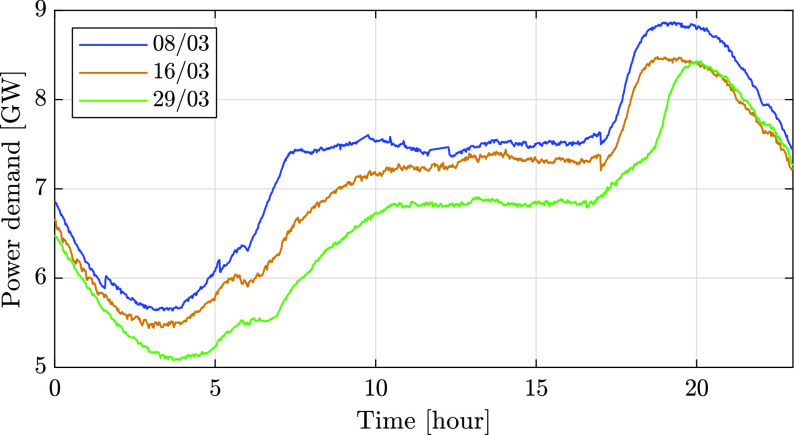
Comparison of the total demand [GW] in three different days during the COVID-19 pandemic in Israel.

**FIGURE 2.**
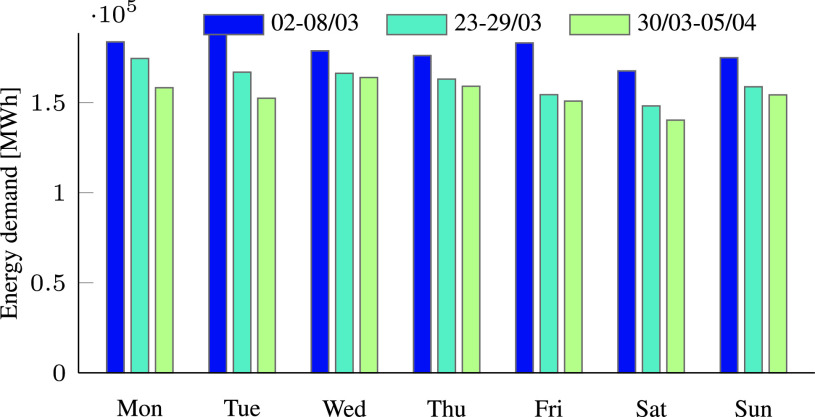
Comparison of the total energy demand [MWh] in three different weeks during the COVID-19 pandemic in Israel.

**FIGURE 3.**
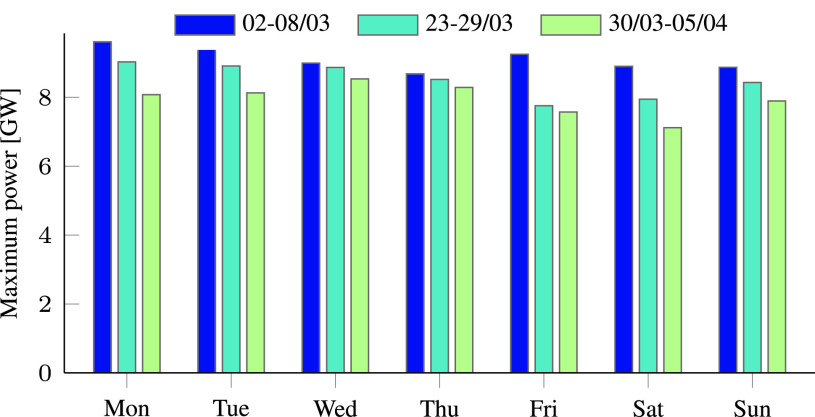
Comparison of the maximum power [GW] in three different weeks during the COVID-19 pandemic in Israel.

The reduced electricity consumption observed during the pandemic is the result of government measures against the virus spread. As policy actions to mitigate COVID-19 became more severe, the consumption decreased. This is demonstrated in Fig. 1, which compares the electricity demand in Israel before and during the COVID-19 pandemic. The figure shows the electricity consumption during three days with the same average temperature of 21 °C. The blue graph is the demand on March 8, before emergency state was declared, the orange graph is the demand on the March 16, when partial restrictions were enforced, and the green graph is the demand on March 29, 10 days after the declaration of a state of emergency. Although the average temperature was the same, substantially reduced consumption was measured from day to day. The same trend may be observed in Table 1 and Fig. 2, which depict the total energy consumption per day for each week, and also in Table 2 and Fig. 3 that present the daily peak power. Both the total energy consumption and peak power continually decreased during March 2020.

**TABLE 1:** Comparison of the Total Energy Demand [MWh] Per Week During COVID-19 Pandemic in Israel

Day/Week	02–08/03	23–29/03	30/03–05/04
Monday	183734	174558	158303
Tuesday	188664	166931	152433
Wednesday	178797	166285	163969
Thursday	176150	163086	159136
Friday	183171	154412	150905
Saturday	167654	148177	140321
Sunday	174928	158820	154338
Total	1253098	1132269	1079406

**TABLE 2:** Comparison of the Maximum Power [MW] Per Day During COVID-19 Pandemic in Israel

Day/Week	02–08/03	23–29/03	30/03–05/04
Monday	9612	9031	8079
Tuesday	9857	8910	8131
Wednesday	8992	8867	8534
Thursday	8681	8519	8288
Friday	9249	7756	7575
Saturday	8903	7945	7119
Sunday	8870	8432	7895
Average	9166	8494	7946

**FIGURE 4.**
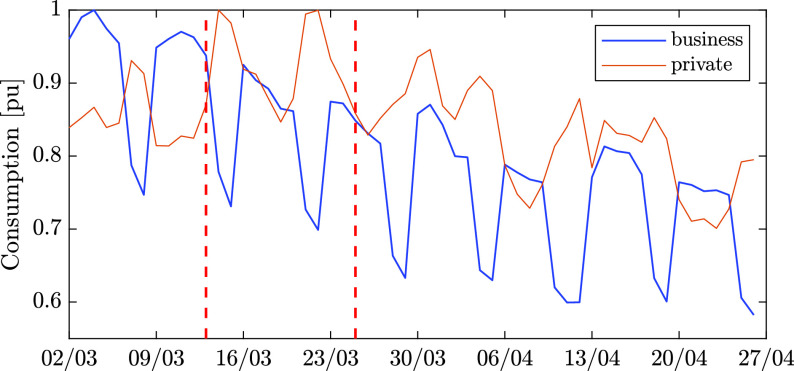
Scaled daily consumption from March 2 to April 26 in Estonia, for business (blue) and private (orange) clients.

**FIGURE 5.**
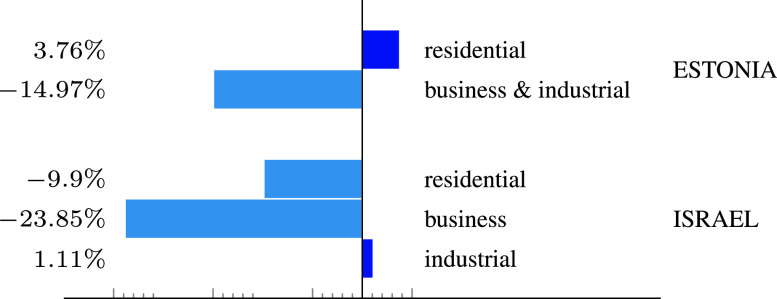
Relative change in the overall weekly consumption between 02-08/03 and 30/03-05/04 in Estonia and Israel.

The daily aggregated electricity consumption in Estonia shows somewhat similar patterns. Figure 4 shows the aggregated consumption for 8 weeks, from March 2 to April 26. In the figure the first vertical dashed line marks the onset of emergency state in Estonia, whereas the second line marks the closure of large shopping malls, restaurants, entertaining facilities, etc. As can be seen in the graph, electricity consumption in the private sector (orange solid line) does not show a consistent pattern of decrease or increase, whereas consumption in the business sector (blue solid line) experienced a gradual decline of approximately 20% during only eight weeks. This data is somewhat surprising, taking into account that home isolation can potentially cause a significant increase in residential electricity consumption. However this trend may be explained by the comfortable weather during the peak of the pandemic, which did not require use of air conditioners or electric heaters. Figure 5 shows the relative change in the overall weekly consumption in several sectors of Estonia and Israel between 02-08/03 and 30/03-05/04. Unlike Estonia, the Israeli residential sector experienced a significant decrease in energy consumption during this period. This can be partly explained by the shifted waking time of people, due to the closure of work places, universities, and schools as well as by the daylight saving time that started on March 27. Both reasons mainly affect lighting energy consumption and heating in the early morning. At the same time, the consumption in the Israeli business sector experienced a dramatic decrease from the same reasons (closure of large facilities, as detailed above). The Israeli industrial sector was barely affected by the pandemic. A possible conclusion is that power grids with many business consumers may be more vulnerable to pandemics and other unusual events.

**FIGURE 6.**
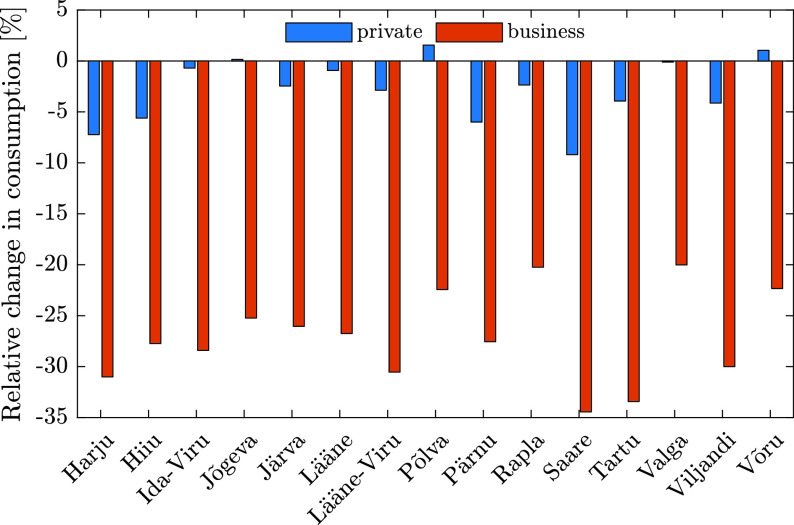
Estonian county-based relative change in consumption for private and business clients from March 2 to April 12.

**FIGURE 7.**
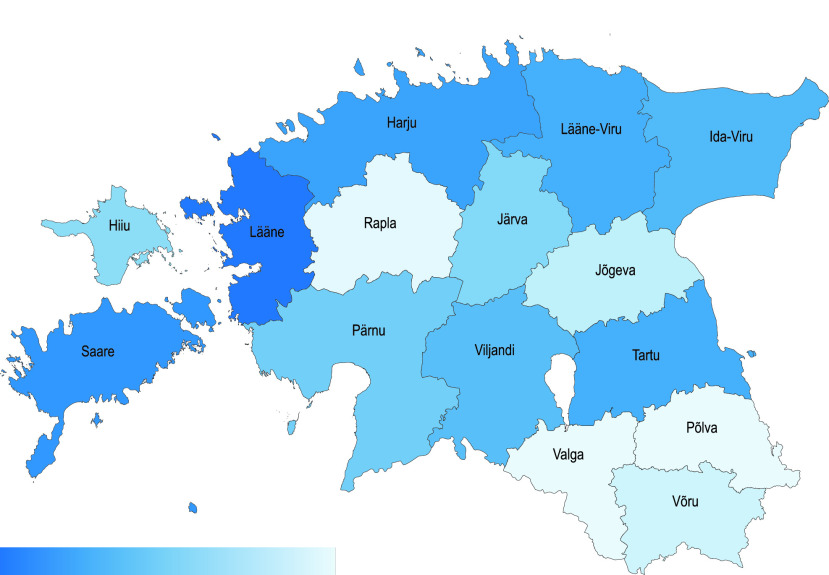
Estonian county-based relative change in total consumption (business and private clients together). The color gradient is in the range [−27%, −14%].

**FIGURE 8.**
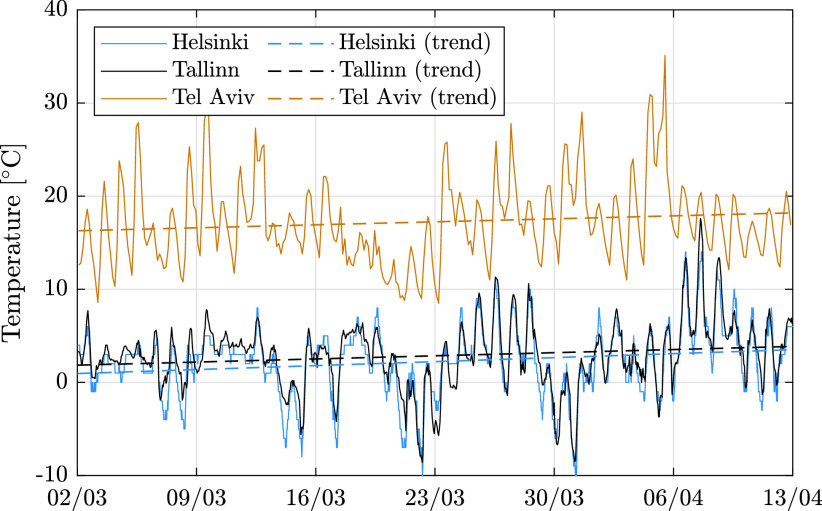
Temperature variation during March 2 to April 12 in Helsinki, Tallinn, and Tel Aviv.

The decrease in electricity consumption in Estonia differs among regions of the country, depending on the unique characteristics of each region. Two significant factors are the presence of industry and commercial businesses, and the severity of COVID-19 virus infection. Figures 6 and 7 illustrate the relative change^1^ in consumption, between March 2 and April 12 in different regions of Estonia. As can be seen in Fig. 7, the counties that experienced the most significant reduction in consumption are the northern regions and Tartu, which are industrial and heavily populated. The consumption ratio in these regions between private and business consumers is just few times shifted toward business clients, but in places like Lääne the shift is more dramatic (up to 80 times at specific dates). Moreover, the most significant reduction, more than 30%, occurred in Saare county, which does not have much heavy industry. This can be explained by the level of the spread-per-person measure (164.9 per 10000 people) in this region, which is more than 8 times higher compared to other regions [14]. Furthermore, between March 2 and April 12 most regions in Estonia experienced an overall reduction in electricity consumption in the private sector. This can be partly explained by the rise in temperature during these dates, as can be seen in Fig. 8, since the average temperature in Tallinn increased by 2–3 °C during this period. Yet this data cannot explain a reduction of 7% in Harju region, or 10% in Saare. One possible explanation for the exceptional reduction in these regions is an overall reduced activity of the citizens, while another explanation is migration of people from large cities to small cities and villages.^1^The relative change is computed as the difference between the start and end points of a trend line $y=\alpha t+\beta $, in which $y$ marks consumption, and $\alpha $, $\beta $ are chosen such that $\sum _{t}(y_{t}-(\alpha t+\beta))^{2}$ is minimized.

**FIGURE 9.**
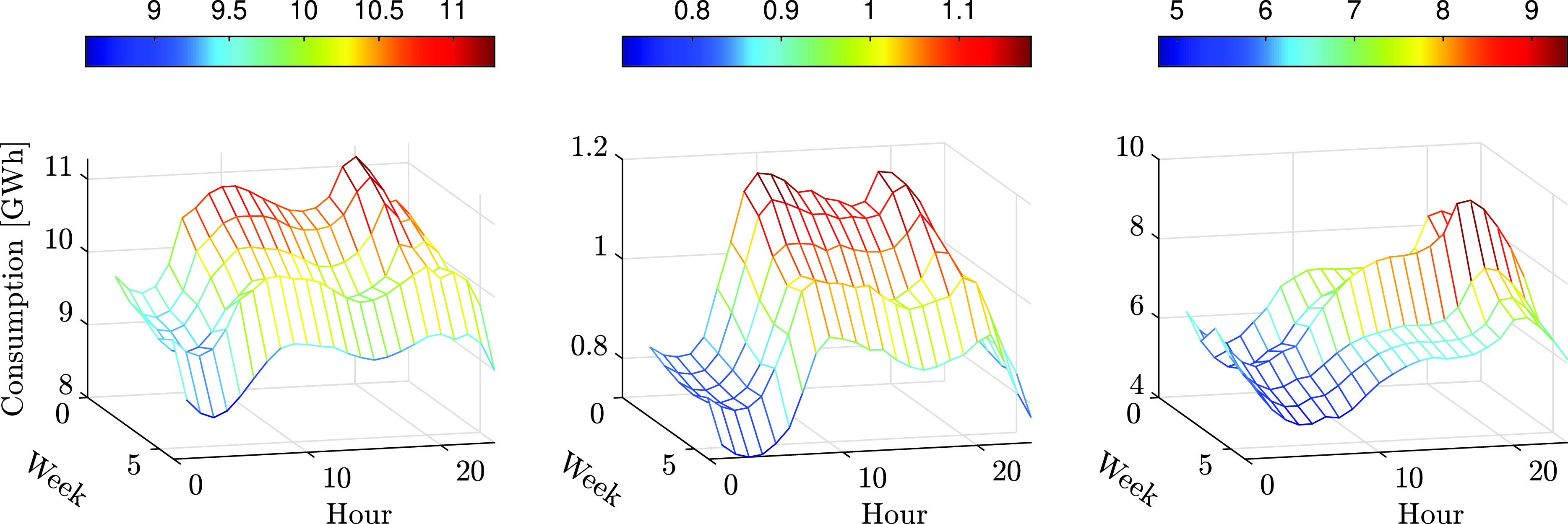
Average total hourly consumption [GWh] in Finland (left plot), Estonia (middle plot), and Israel (right plot) from March 2 to April 12.

**FIGURE 10.**
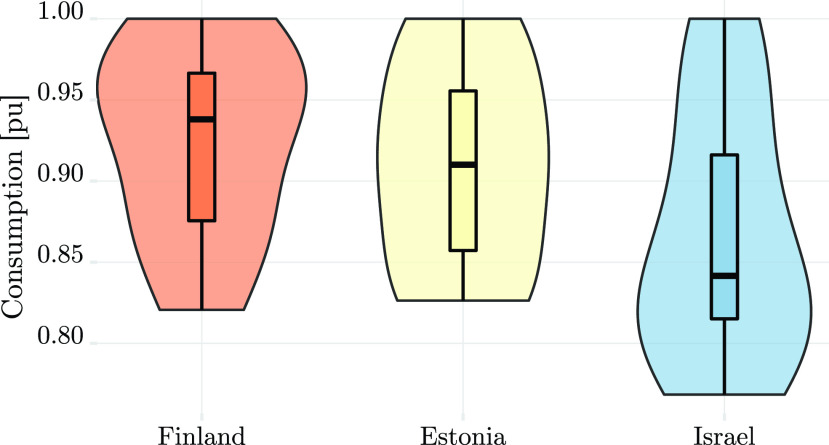
Hourly electricity consumption distributions during March 2 to April 26.

**FIGURE 11.**
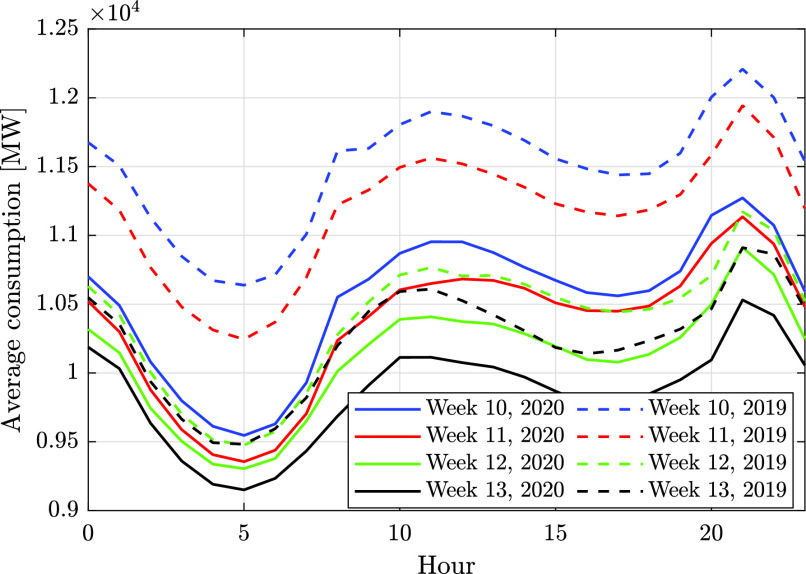
Total average hourly consumption in Finland, March 2019/2020.

**FIGURE 12.**
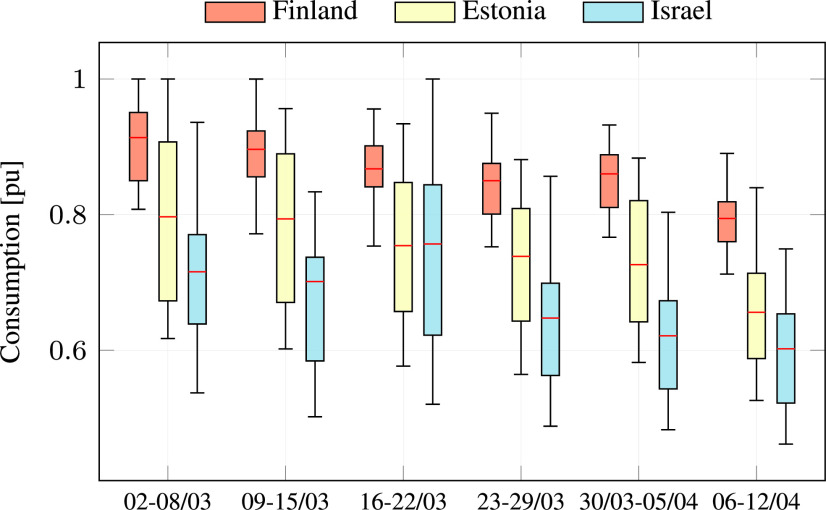
Consumption statistics over the period March 2 to April 12.

In addition to reducing the overall energy consumption, the COVID-19 virus also changed patterns of consumption along the day, causing a more “smooth” consumption curve. Figure 9 focuses on the consumption in all countries between March 2 and April 12, showing that as time progresses the consumption becomes more smooth from week to week. This may be the result of diversification in the waking time of people. The spike during the third week of March in Israel can be explained by the weather, since the mean temperature dropped to 13.9 °C during the week, compared to 18.2 and 18 °C in the previous and subsequent weeks (see Fig. 8). Figure 10 depicts the scaled distribution of consumption over an 8 weeks horizon. The scaling factors are selected to be the peak-power in each country during the considered period: 11668 MW in Finland, 1304 MW in Estonia, and 9972 MW in Israel. Although the patterns are slightly different due to the delay in the announcement of emergency state and the local weather, the end result is similar: while Estonia and Finland experienced a smooth and gradual decrease in consumption, the Israeli grid experienced a larger variation in consumption, as illustrated in the distribution shown in Fig. 10. A possible explanation can be the relatively high share of business consumers in Israel (~28%), which were largely shut down during the pandemic, as seen in Fig. 5. In addition, Fig. 11 compares the average hourly consumption in Finland during March in both 2019 and 2020, showing that the consumption curve in March 2020 is smoother than in 2019, and this phenomenon strengthens from week to week as the number of people in quarantine increases. This can also be explained from a statistical point of view as shown in Fig. 12, which presents box plots of the scaled hourly consumption in Finland, Estonia, and Israel. For each box the red line is the median, and the bottom and top edges are the 25th and 75th percentiles, respectively. The whiskers extend to the most extreme data points. As time progresses the graph clearly shows that the demand curve becomes more flat, since the boxes become smaller.

**FIGURE 13.**
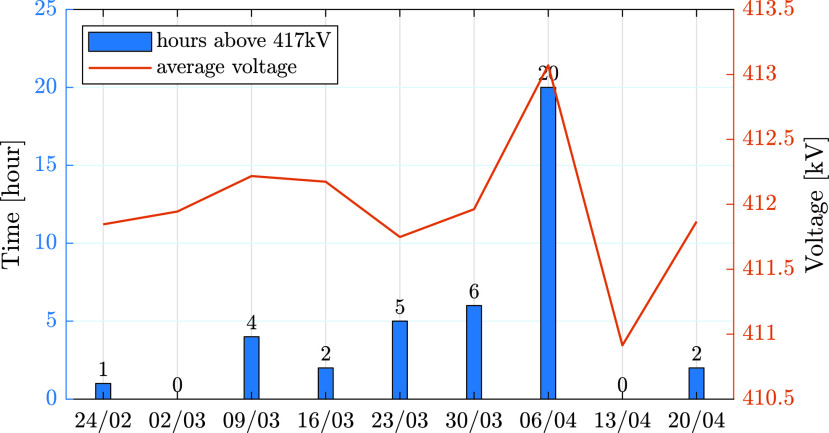
Average voltage (orange) and total hours above 417 kV (blue) in the 400 kV transmission network in Israel.

The reduced consumption due to the pandemic also led to significant voltage deviations. This is due to capacitive elements in transmission lines that generate reactive power. When the overall consumption is low, this reactive power changes the power flow in the network, and results in increased voltage amplitudes. This phenomena is demonstrated in Fig. 13, which presents the number of hours in which the average voltage in the 400 kV transmission system in Israel increased above 417 kV. The data is presented for 9 weeks, from February 24 until April 26. As can be seen in the graph, the amount of time in which the voltage was higher than 417 kV increases consistently from the end of February until the beginning of April. The increase in voltage levels during March can be explained by the reduced consumption, which is depicted in Fig. 1. The spikes observed in the graph during the week of April 6 is the result of the Passover holiday, during which the consumption was extremely low. The decline in voltage after this week can be explained by less strict measures that were initiated by the Israeli government after Passover, which led to increased demand.

## III. Frequency Stability and the Effects of Renewable Energy Sources

In this section we attempt to explain how low power consumption during the pandemic affected the frequency stability of the Israeli and Finnish grids. Specifically, we show that in the Israeli grid the low consumption during the pandemic caused significant deviations of the frequency from its nominal value. However, this was not the case in the Finnish grid, probably since Finland trades significantly more power with its neighbors (most prominently Russia, Sweden, and Estonia) [15]. Moreover, we show that the low consumption raised the relative share of renewable energy sources in Israel, which caused a number of adverse effects:

• Reduced spinning reserves, and therefore poor resiliency;
• Increased ramp-rates of conventional power;
• Reduced rotational inertia, which may cause increased frequency deviations;
• Rapid changes in conventional power generation, which may lead to poor reliability, and non-optimal economic dispatch.

**FIGURE 14.**
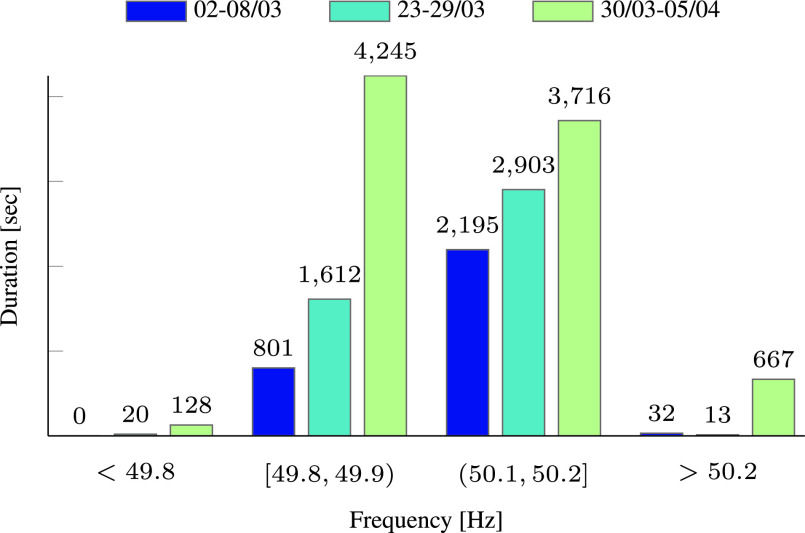
Duration [in sec] of all frequency deviations—comparison of three different weeks before/during the COVID-19 pandemic in Israel.

The effects of the COVID-19 pandemic on the frequency of the Israeli grid are summarized in Tables 3, 4, and 5. These tables present the number of seconds in which the frequency deviated from its nominal value during each day, for three weeks in March and April. During all weeks reported the average temperature and clouds motion were similar, to mitigate the effects of varying weather conditions. The frequency deviations are divided to higher or lower than 100 mHz and 200 mHz. The same data is summarized in Fig. 14, which presents the overall frequency deviations per week. As can be seen in this figure, the overall duration of high frequency deviations increases as time progresses. This can be explained by the decrease in consumption, which raised the relative share of solar generation in Israel, and led to more frequent events of energy imbalance.

**TABLE 3:** Daily Based Duration [in Sec] of Frequency Deviations in Israel During March 2 to March 8

Day	< 49.8	[49.8,49.9)	(50.1,50.2]	>50.2
Monday	0	42	49	1
Tuesday	0	13	569	2
Wednesday	0	42	1	1
Thursday	0	121	591	8
Friday	0	415	100	9
Saturday	0	7	80	1
Sunday	0	161	805	10
Total	0	801	2195	32

**TABLE 4:** Daily Based Duration [in sec] of Frequency Deviations in Israel During March 23 to March 29

Day	< 49.8	[49.8, 49.9)	(50.1,50.2]	>50.2
Monday	0	0	137	1
Tuesday	0	1	40	1
Wednesday	20	429	293	2
Thursday	0	400	187	1
Friday	0	362	876	6
Saturday	0	406	1054	1
Sunday	0	14	316	1
Total	20	1612	2903	13

**TABLE 5:** Daily Based Duration [in Sec] of Frequency Deviations in Israel During March 30 to April 5

Day	< 49.8	[49.8, 49.9)	(50.1,50.2]	>50.2
Monday	1	495	485	46
Tuesday	1	544	277	190
Wednesday	1	147	244	19
Thursday	1	745	255	7
Friday	121	961	1149	82
Saturday	1	590	637	174
Sunday	1	763	669	149
Total	128	4245	3716	667

**FIGURE 15.**
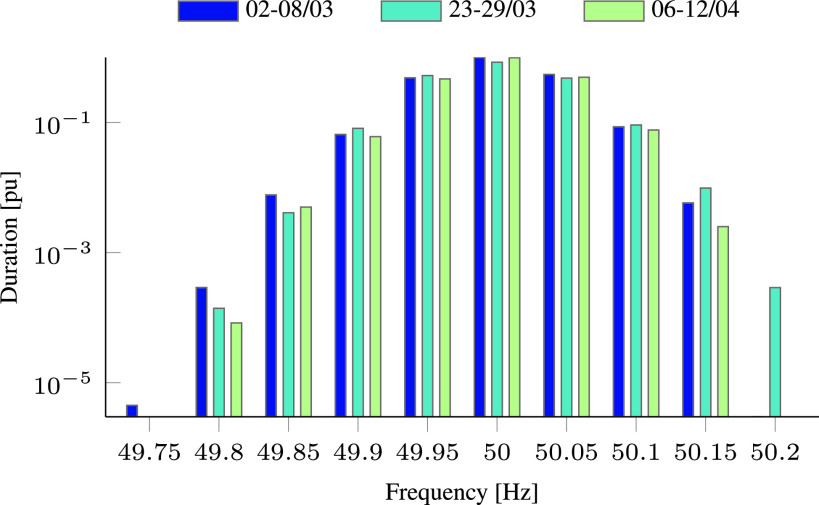
Scaled time of frequency deviations—comparison of three different weeks before/during the COVID-19 pandemic in Finland.

Figure 15 presents the aggregated (to ±0.02 Hz intervals) duration of frequency deviations during three different weeks before and during the COVID-19 pandemic in Finland. Here we scaled the overall duration and used a semi-logarithmic scale to visualize the small number of low and high frequency events. As can be seen, the duration of frequency deviations does not follow a clear pattern, in spite of the reduced consumption shown in Fig. 12. The different frequency response in Israel and Finland can be explained by the fact that Israel is an energy island, while Finland is connected to the strong grids of Sweden and Russia.

**FIGURE 16.**
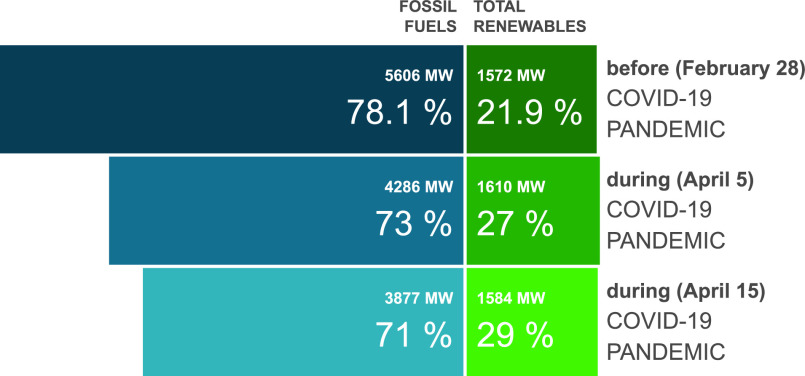
Maximum share of renewable energy in Israel, as a fraction of total generation.

Another impact of the COVID-19 pandemic and the low consumption it caused is an increase in the relative share of renewable energy sources. Due to various economical and environmental reasons renewable energy sources are often prioritized over conventional power plants. As a result, when consumption is low, the relative share of renewables in the generation mix increases. This phenomena is demonstrated in Fig. 16, which presents the maximal share of renewable energy before and during COVID-19 pandemic in Israel. Before the pandemic the maximum fraction of renewable energy in Israel was 21.9%. However, on April 4 at 12:55 the solar share reached to 27% of the total generation, the maximum fraction of renewable energy ever measured in Israel. This record was broken again on April 5, which was a holiday, in which the solar share reached 29%. As can be seen in Fig. 16, in all cases the generated solar power was almost equal, and so the reason for the high share of renewable energy is the low consumption.

**FIGURE 17.**
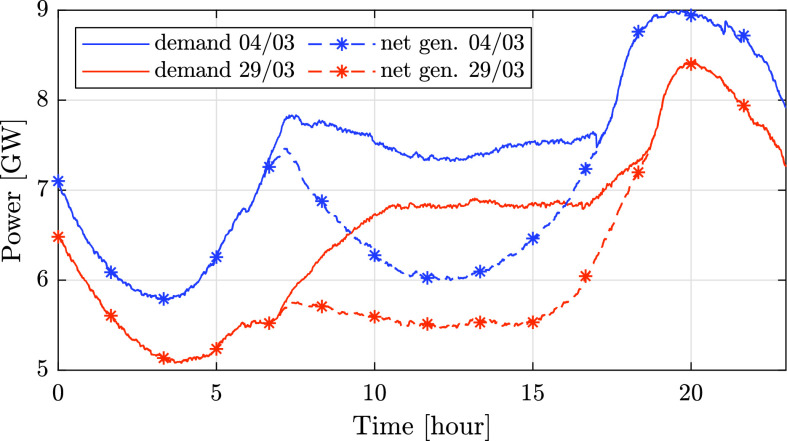
Comparison of the total demand and conventional generation during 24 hours on two sunny days: March 4 and March 29.

A high share of renewable energy alongside low consumption may cause increased ramp-rates of conventional power, which may lead to reduced reliability, poor resiliency, and non-optimal economic dispatch. To demonstrate this, in Fig. 17 we compare the overall demand and conventional generation in Israel during 24 hours in both March 4 and 29. As seen in the graph, the consumption on the 29th is substantially lower, resulting in a 20% share of renewable energy, in comparison to 17.5% on the 4th. Correspondingly, the ramp-rate of conventional generation between 15:00 and 20:00 on the 29th is significantly higher, with an increase of approximately 2900 MW, which was 34% of the peak conventional generation on that day, in comparison to an increase of about 2500MW on the 4th, which was 27% of the peak conventional generation. Additionally, the high share of renewable energy on these days led to increased frequency deviations. This can be seen in Tables 3 and 4, which show that the overall duration of frequency deviations on the 29th is 331 seconds, as opposed to only 44 seconds on the 4th. This can be explained by the reduced rotational inertia and spinning reserves, which decrease the frequency stability and limit the ability of the system operator to address events of energy imbalance.

**FIGURE 18.**
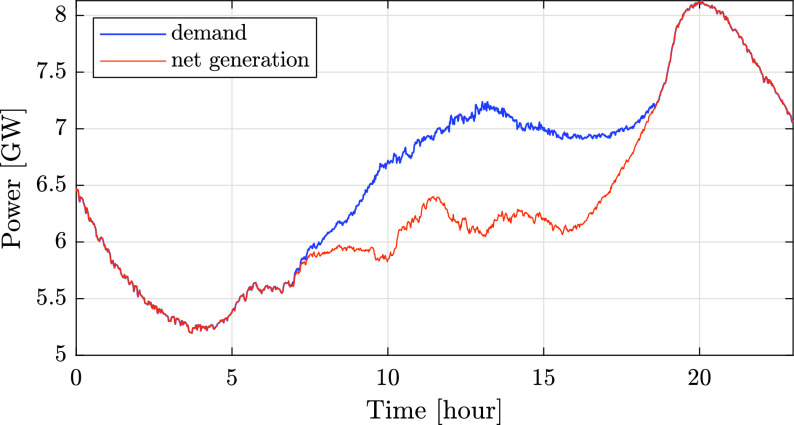
Comparison of the total demand and conventional generation [GW] during 24 hours on March 31 (cloudy day).

**FIGURE 19.**
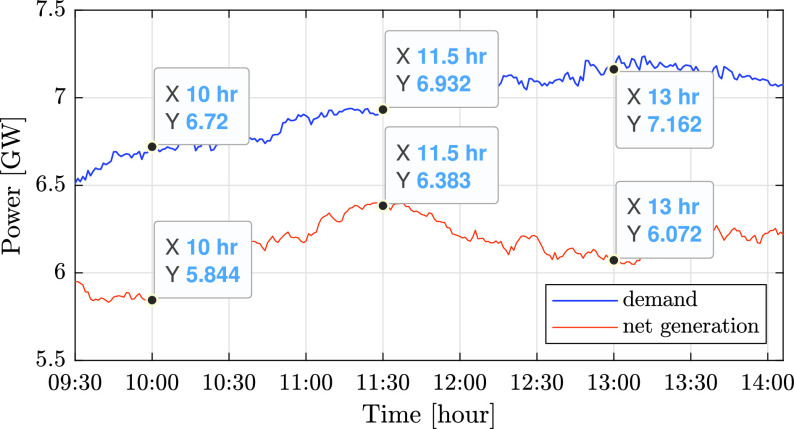
Comparison of the total demand and conventional generation [GW] between 9:30 and 14:00 on March 31.

An additional effect of the low consumption was rapid changes in conventional power, due to intermittent solar generation. This effect is demonstrated for the Israeli grid in Figs. 18 and 19, which present both the overall demand and conventional generation on a cloudy day (March 31). In both graphs the difference between the demand and conventional generation is the renewable generation. Figure 18 shows the data during 24 hours, while Fig. 19 focuses on times of peak solar power, between 9:30 and 14:00. As can be seen in Fig. 18, intermittent solar photovoltaic generation, caused by the motion of clouds, leads to rapid changes in conventional generation. More specifically, Fig. 19 shows that the solar fraction of the overall power fluctuated from 13% at 10:00 to 7.9% at 11:30 and 15.2% at 13:00, causing changes in conventional generation accordingly.

## IV. Lessons From the COVID-19 Pandemic

A main lesson to be learned is that the low consumption triggered by the COVID-19 pandemic is a major challenge. Abnormally low electricity consumption affects the management and control of generation units, and as demonstrated in the results above, may lead to a reduced system reliability and resiliency. When electricity consumption is exceptionally low, the system operator has to shut-down conventional power plants that are normally operated, until consumption goes back to normal. Moreover, in countries with a high share of solar generation, several conventional power plants have to be operated in a two-shift operation mode. In this mode, units are shut-down during the morning, when solar generation rises, and are synchronized back to the grid in the afternoon, when solar generation declines. This daily routine causes several problems:

1) Several types of conventional power plants that are shut-down for a few days cannot be fully activated in a short time. This means that in case of contingency, such as a failure in a generation unit or an unexpected load deviation, backup units with a short startup time are used. These units are typically expensive and polluting.
2) The two-shift operation mode of power plants may result in equipment deterioration and higher maintenance costs. Furthermore, there may be delays in the synchronization of these units to the grid, making it difficult for the system operator to supply the evening demand.
3) Low demand leads to high voltages in the transmission network. Usually, this problem is solved by operating synchronous machines in an under-excited mode, in which they absorb reactive power. However, when less power plants are being used, the ability to absorb reactive power and control the voltage is reduced.
4) Less operating power plants means less spinning reserve and lower rotational inertia. Therefore the frequency response is limited, and any small deviation in renewable energy production may cause stability issues. Moreover, in case of failure such as a transmission line short-circuit or a unit malfunction, the change in frequency is sharper and faster, which can lead to additional problems.

Table 6 directly compares the main characteristics of the three systems considered in this research (Finland, Estonia, and Israel), emphasizing the relative reduction in consumption due to the pandemic, and the approximate number of significant frequency events. The data shows clearly the effects of interconnections on the stability and reliability of the network. For instance, it can be seen that the Estonian grid did not have a significant number of frequency events during the first weeks of the COVID-19 pandemic, while the Israeli grid shows a significant increase in the number of such events, as a result of the low consumption. At the same time the Finnish grid does not exhibit a clear pattern. The fact that Israel had more frequency events than Finland can be explained by the fact that Finland is interconnected to larger networks, whereas the Israeli grid is effectively an island. The same conclusions may apply to other systems: grids that are either large or interconnected to other large systems will probably be more resilient to unusual events such as the COVID-19 pandemic. Another important factor may be the relative share of renewable sources: grids with a lower relative share of renewable sources will probably be more resilient to events of low consumption.

**TABLE 6:** Main Characteristics of the Finnish, Estonian, and Israeli grids

Characteristics	Finland	Estonia	Israel
Population size [mln]	5.52	1.32	9.21
Total area [km^2^]	338424	45227	22072
Emergency state announcement	March 16	March 12	March 19
Grid nominal frequency [Hz]	50		
Details of grid connection	AC connections to Sweden and Russia; DC links to Estonia	Part of BRELL power grid; 2 DC connections to Finland	Islanded
Total installed generation capacity [GW]	17.4 as of 2017	2.907 as of 2018	18.35 as of 2019
All-time peak consumption [GW]	15.01	1.587	13.568
Total consumption 2019 [GWh]	83.439	8.23	68.487
Total production 2019 [GWh]	63.802	6.098	72.426
Total consumption March, 2020 [GWh]	7593	742	5392
Total consumption April, 2020 [GWh]	6584	640	4496
Share of renewables (2019) [in %]	38.7	28.6	7
Relative total consumption reduction [in %], (28/02 vs 05/04)	19.1	27.9	17
Duration [in sec] of significant (±0.2 Hz) frequency events at the first/last weeks of March	No clear pattern	Stable operation	32/795

The problems mentioned above emphasize that system operators need additional tools to manage events of abnormally low electricity consumption, and that in such events operational considerations should be prioritized over economical ones [16], [17]. In times of crisis, human behavior is hard to predict, and so is energy consumption. Therefore, the system operator needs more operational reserves than usual. If a unit is stopped due to low consumption, economic considerations dictate that the most expensive units should be turned-off first. Yet, this might leave the system operator with a set of units that do not supply the operational capabilities needed to maintain the stability and flexibility of the system. Therefore, one possible conclusion from the COVID-19 pandemic is that during periods of low consumption unit commitment planning procedures should promise enough flexibility, which will be measured by a combination of good inertial response, high ramp rate, large spinning reserve, and a large range in power output.

In addition, once the COVID-19 pandemic is completely under control, a rigorous analysis of the measurements that were taken to maintain the stability of power systems worldwide should be done. Such an analysis can be used to enrich existing emergency plans, which currently mainly address events such as earthquakes or extreme weather, with the appropriate steps to be taken during a health crisis. Such emergency plans should also include means for estimation and control. For example, system operators should conduct daily meetings in order to discuss the problems that arise, and the appropriate means to address them.

To deal with future pandemics, ancillary services that enhance the flexibility of power systems, such as operating reserves and voltage control, should be further encouraged through appropriate pricing schemes. The COVID-19 pandemic showed that such services are more important than ever before. There are many technological innovations that can enrich the toolbox of a grid operator, however existing regulations do not always support their usage. For example, at certain times during the pandemic, the operating units in Israel did not provide enough spinning reserve, and so the frequency of the system was at risk. The main reason for the lack of spinning reserve is the absence of any regulatory compensation that may encourage it.

Furthermore, the pandemic provided an interesting opportunity to experience higher relative shares of renewable energy in power grids, since lower demand for electricity is equivalent to a higher share of renewable energy sources. The information gathered during this event can be used by decision makers to either update renewable energy targets, or to improve the readiness of their grids to reach the existing goals on schedule. Perhaps in the long run the COVID-19 pandemic can boost the integration of renewable energy, since the needed tools for dealing with low consumption during a pandemic are the same as the ones needed to support more renewable sources. These tools include energy storage devices, enhanced control of variable renewable energy sources, and frequency regulation capabilities [18]–[20].

## V. Conclusions

This paper explores how the COVID-19 pandemic affected the operation of small electric grids, and what can this event teach us on the readiness of such grids in the face of future global health crises. One main conclusion is that the reduced energy consumption during the pandemic is critical, and has a major effect on the operation of the grid. Abnormally low electricity consumption affects the management and control of generation units, and as demonstrated in the results above, may lead to voltage and frequency deviations, and more generally to reduced reliability and resiliency. We argue that system operators need additional tools to manage events of abnormally low electricity consumption, such as storage devices, enhanced control of variable renewable energy sources, and frequency regulation capabilities. Since flexibility is critical we also conclude that in many cases long-term reliability should be preferred over immediate economical considerations. Furthermore, to deal with future pandemics, ancillary services that enhance the flexibility of power systems, such as operating reserves and voltage control, should be further encouraged through appropriate pricing schemes. These conclusions are especially relevant for small and isolated grids. The pandemic also pushed renewable energy to record highs, hence the data gathered during this event may help clarify the influence of a high share of renewables on electric grids, and can offer us a glance into a renewable-rich future.

## References

[1] D. S. Hui, E. I. Azhar, T. A. Madani, F. Ntoumi, R. Kock, O. Dar, G. Ippolito, T. D. Mchugh, Z. A. Memish, C. Drosten, A. Zumla, and E. Petersen, “The continuing 2019-nCoV epidemic threat of novel coronaviruses to global health—The latest 2019 novel coronavirus outbreak in Wuhan, China,” Int. J. Infect. Dis., vol. 91, pp. 264–266, Oct. 2020, doi: 10.1016/j.ijid.2020.01.009. 31953166 PMC7128332

[2] L. Peng, W. Yang, D. Zhang, C. Zhuge, and L. Hong, “Epidemic analysis of COVID-19 in China by dynamical modeling,” medRxiv, vol. 2018, pp. 1–18, Feb. 2020, doi: 10.1101/2020.02.16.20023465.

[3] Y.-Y. Zheng, Y.-T. Ma, J.-Y. Zhang, and X. Xie, “COVID-19 and the cardiovascular system,” Nature Rev. Cardiology, vol. 17, no. , pp. 259–260, 5 2020, doi: 10.1038/s41569-020-0360-5. 32139904 PMC7095524

[4] World Health Organization. Statement on the Second Meeting of the International Health Regulations (2005) Emergency Committee Regarding the Outbreak of Novel Coronavirus (2019-nCoV). Accessed: 5 8, 2020. [Online]. Available: https://www.who.int/news-room/detail/30-01-2020-statement-on-the-second-meeting-of-the-international-health-regulations-(2005)-emergency-committee-regarding-the-outbreak-of-novel-coronavirus-(2019-ncov)

[5] F. Birol. (2020). The Coronavirus Crisis Reminds us That Electricity is More Indispensable Than Ever. Accessed: Apr. 9, 2020. [Online]. Available: https://www.iea.org/commentaries/the-coronavirus-crisis-reminds-us-that-electricity-is-more-indispensable-than-ever

[6] P. Bhagwat. (2020). Identifying Impacts of COVID-19 on the Indian Power Sector. Accessed: Apr. 9, 2020. [Online]. Available: https://fsr.eui.eu/identifying-impacts-of-covid-19-on-the-indian-power-sector/

[7] G. Wilson, N. Godfrey, S. Sharma, and T. Bassett. (2020). Here’s How Energy Demand Has Changed During the UK’s Lockdown. Accessed: Apr. 9, 2020. [Online]. Available: https://www.weforum.org/agenda/2020/04/we-analysed-electricity-demand-and-found-coronavirus-has-turned-weekdays-into-weekends/

[8] W. de Freitas. (2020). COVID-19: Will Slow the Global Shift to Renewable Energy, But Can’t Stop it. Accessed: Apr. 9, 2020. [Online]. Available: https://theconversation.com/covid-19-will-slow-the-global-shift-to-renewable-energy-but-cant-stop-it-133499

[9] S. Shah. (2020). COVID-19: Clean Energy Challenges and Opportunities. Accessed: Apr. 9, 2020. [Online]. Available: https://www.sc.com/en/trade-beyond-borders/covid-19-clean-energy-challenges-and-opportunities/

[10] K. Das. (2020). Impact of COVID-19 Pandemic Into Solar Energy Generation Sector. Accessed: 5 5, 2020. [Online]. Available: https://ssrn.com/abstract=3580341

[11] B. Marandi, J. Dunleavy, G. S. Hamilton, R. Morrison, M. Collman, and J. Corrigan. (2020). COVID-19: What it Means for the Energy Industry. Acessed: Apr. 9, 2020. [Online]. Available: https://www.pwc.com/us/en/library/covid-19/coronavirus-energy-industry-impact.html

[12] C. Albulescu, “Coronavirus and oil price crash,” SSRN Electron. J., vol. 15, pp. 1–13, Oct. 020, doi: 10.2139/ssrn.3553452.

[13] A. Atkeson. (2020). What Will be the Economic Impact of COVID-19 in the US? Rough Estimates of Disease Scenarios. Accessed: Jun. 29, 2020. [Online]. Available: https://www.nber.org/papers/w26867.pdf

[14] Terviseamet. (2020). Koroonaviiruse Andmestik. Accessed: 5 9, 2020. [Online]. Available: https://www.terviseamet.ee/et/koroonaviirus/koroonakaart

[15] J. Jääskeläinen, S. Höysniemi, S. Syri, and V.-P. Tynkkynen, “Finland’s dependence on russian energy—Mutually beneficial trade relations or an energy security threat?” Sustainability, vol. 10, no. 10, p. 3445, Sep. 2018, doi: 10.3390/su10103445.

[16] A. Navon, P. Kulbekov, S. Dolev, G. Yehuda, and Y. Levron, “Integration of distributed renewable energy sources in israel: Transmission congestion challenges and policy recommendations,” Energy Policy, vol. 140, 5 2020, Art. no. 111412, doi: 10.1016/j.enpol.2020.111412.

[17] A. A. Solomon, D. Faiman, and G. Meron, “An energy-based evaluation of the matching possibilities of very large photovoltaic plants to the electricity grid: Israel as a case study,” Ener. Policy, vol. 38, no. 10, pp. 5457–5468, 2010, doi: 10.1016/j.enpol.2009.12.024.

[18] P. Denholm and M. Hand, “Grid flexibility and storage required to achieve very high penetration of variable renewable electricity,” Energy Policy, vol. 39, no. 3, pp. 1817–1830, Mar. 2011, doi: 10.1016/j.enpol.2011.01.019.

[19] B. Kroposki, B. Johnson, Y. Zhang, V. Gevorgian, P. Denholm, B.-M. Hodge, and B. Hannegan, “Achieving a 100% renewable grid: Operating electric power systems with extremely high levels of variable renewable energy,” IEEE Power Energy Mag., vol. 15, no. 2, pp. 61–73, Mar. 2017, doi: 10.1109/MPE.2016.2637122.

[20] F. Milano, F. Dorfler, G. Hug, D. J. Hill, and G. Verbic, “Foundations and challenges of low-inertia systems (Invited Paper),” in Proc. Power Syst. Comput. Conf. (PSCC), Jun. 2018, pp. 1–25, doi: 10.23919/PSCC.2018.8450880.

